# Environmental Enrichment Components Required to Reduce Methamphetamine-Induced Behavioral Sensitization in Mice: Examination of Behaviors and Neural Substrates

**DOI:** 10.3390/jcm11113051

**Published:** 2022-05-28

**Authors:** Cai-N Cheng, Shaw-Jye Wu, Andrew Chih Wei Huang

**Affiliations:** 1 Department of Life Sciences, National Central University, Jhong-Li District, Taoyuan City 32001, Taiwan; shps90060801@yahoo.com.tw; 2 Department of Psychology, Fo Guang University, No. 160, Linwei Road, Jiaosi Shiang, Yilan City 26247, Taiwan

**Keywords:** environmental enrichment, behavioral sensitization, methamphetamine, medial prefrontal cortex, amygdala, hippocampus, nucleus accumbens, mice

## Abstract

Environmental enrichment (EE) involves the presentation of various sensory, physical, social, and cognitive stimuli in order to alter neural activity in specific brain areas, which can ameliorate methamphetamine (MAMPH)-induced behavioral sensitization and comorbid anxiety symptoms. No previous studies have comprehensively examined which EE components are critical for effectively reducing MAMPH-induced behavioral sensitization and anxiety. This study examined different housing conditions, including standard housing (SH, No EE), standard EE (STEE), physical EE (PEE), cognitive EE (CEE), and social EE (SEE). In the beginning, mice were randomly assigned to the different combinations of housing conditions and injections, consisting of No EE/Saline, No EE/MAMPH, STEE/MAMPH, PEE/MAMPH, CEE/MAMPH, and SEE/MAMPH groups. Then, the mice received intraperitoneal injections of 1 mg/kg MAMPH or normal saline daily for 7 days, followed by a final injection of 0.5 mg/kg MAMPH or normal saline. After behavioral tests, all mice were examined for c-Fos immunohistochemical staining. The results showed that MAMPH induced behavioral sensitization as measured by distance traveled. MAMPH appeared to induce lowered anxiety responses and severe hyperactivity. All EE conditions did not affect MAMPH-induced lowered anxiety behaviors. STEE was likely more effective for reducing MAMPH-induced behavioral sensitization than PEE, CEE, and SEE. The c-Fos expression analysis showed that the medial prefrontal cortex (i.e., cingulate cortex 1 (Cg1), prelimbic cortex (PrL), and infralimbic cortex (IL)), nucleus accumbens (NAc), basolateral amygdala (BLA), ventral tegmental area (VTA), caudate-putamen (CPu), and hippocampus (i.e., CA1, CA3, and dentate gyrus (DG)) contributed to MAMPH-induced behavioral sensitization. The Cg1, IL, NAc, BLA, VTA, CPu, CA3, and DG also mediated STEE reductions in MAMPH-induced behavioral sensitization. This study indicates that all components of EE are crucial for ameliorating MAMPH-induced behavioral sensitization, as no individual EE component was able to effectively reduce MAMPH-induced behavioral sensitization. The present findings provide insight into the development of non-pharmacological interventions for reducing MAMPH-induced behavioral sensitization.

## 1. Introduction

Behavioral sensitization is commonly associated with chronic or repeated use of psychostimulants (e.g., amphetamines or cocaine) resulting in neuroadaptation [1,2]. Some doses of methamphetamine (MAMPH) are known to cause locomotor sensitization in mice [3], although long-term challenge is rarely assessed. Sensitization not only induces behavioral changes but also activates reward pathways mediated by the dopaminergic system in the brain [4]. In addition, this sensitized brain dopamine system probably causes drug cravings and compulsive behaviors in drug use [5].

Environmental enrichment (EE) is defined as a specific housing style that contains rich stimuli designed to activate the sensory, physical, motor activity, and cognitive components of the brain [6]. Different EE styles have been associated with different behavioral, physiological, and biochemical responses [7]. EE can be categorized into standard EE (STEE), which includes all the different stimuli types (e.g., sensory stimulation, physical stimulation, motor activity, and cognitive stimulation) [8,9]; physical EE (PEE), which primarily focuses on physical stimulation and motor activity performed on a running wheel [7,10]; cognitive EE (CEE), which provides a rich cognitive environment, including toys and shelter but without a running wheel [11]; and social EE (SEE), which emphasizes social interactions by increasing the number of companions without providing toys or a running wheel [7,10]. In light of previous evidence, the standard enriched environment could ameliorate behavioral sensitization and anxiety behaviors [8,9]. For example, mice with STEE and continuous exposure to novel objects showed disrupted amphetamine-caused behavioral sensitization and anxiety behaviors, but intermittent exposure to novel objects did not influence behavioral sensitization and anxiety induced by amphetamine [8]. Rats reared in the STEE condition had attenuated behavioral sensitization in locomotor activity compared to rats reared in the isolation or standard housing conditions [9]. This category of studies primarily utilized STEE that included all the different stimuli types, such as sensory stimulation, physical stimulation, motor activity, and cognitive stimulation. Accordingly, these previous studies demonstrated that the STEE effectively interfered with behavioral sensitization and anxiety behaviors.

On the other hand, whether the separate components of EE (e.g., PEE, CEE, or SEE) can modulate behavioral sensitization and anxiety behaviors remains unknown. Little research has demonstrated whether various components of EE seem to regulated distinct behavioral, cognitive, neuronal, and synaptic functions [10,11]. For example, SEE increased prosocial 50-kHz ultrasonic vocalizations and social behaviors, but it did not affect cognitive functions and neuronal plasticity. In contrast, PEE enhanced neurogenesis in the dentate gyrus (DG) of the hippocampus; however, PEE appeared to be worse effects in social approach behaviors [10]. An acute CEE without exercise training improved recognitive function and working memory; moreover, CEE also facilitated neuronal growth factors (e.g., neurotrophins and hippocampal neurogenesis), and the findings indicated that CEE could improve cognitive functions in behavior, neuronal survival, and synaptogenesis in the DG of the hippocampus [11]. The present study employed a variety of EE designs (including STEE, PEE, CEE, and SEE) to examine whether these environments had different effects on MAMPH-induced behavioral sensitization and anxiety behaviors.

Previous studies indicated that EE was able to reduce the symptoms of neurodegeneration diseases, psychiatric diseases, and drug addiction [6,12]. However, data regarding the effects of EE on psychostimulant-induced behavioral sensitization have been inconsistent [13,14]. For example, EE combined with low-dose amphetamine (0.3 mg/kg) blunted the development of amphetamine-induced behavioral sensitization in locomotor activity and hyperactivity; however, EE did not affect dopamine levels in the nucleus accumbens (NAc) or striatum [13]. An enriched environment interfered with cocaine-induced behavioral sensitization; moreover, it was associated with decreases in immediate early gene zif-268 expression in NAc and Delta-Fos B levels in the striatum [15]. Another study reported the opposite effect, in which EE reduced the reward of conditioned place preference but did not affect behavioral sensitization induced by heroin [16], and heroin was found to increase dopamine levels in the NAc [16]. On the other hand, the open-field test has been shown to simultaneously measure locomotor activity and anxiety behavior, covering more functions than the elevated plus-maze task or zero-maze task to singly test anxiety behaviors [17,18]. Therefore, the present study used the open-field task to examine whether EE could attenuate MAMPH-induced behavioral sensitization and comorbid anxiety behaviors.

A growing body of evidence has indicated that the frontal cortex, amygdala, hippocampus, NAc, ventral tegmental area (VTA), and striatum (including caudate nucleus and putamen (CPu)) are involved in EE-mediated modulation of behavioral sensitization [19,20,21,22]. For example, EE has been shown to ameliorate chronic toluene-induced behavioral sensitization and to increase D1 receptors in the prefrontal cortex, NAc, hippocampus, and caudate [22]. Rats housed in EE conditions showed decreased dopamine secretion in the striatum and reduced serotonin levels in the NAc following MAMPH-induced behavioral sensitization [20]. Rats housed in EE conditions also display reduced locomotor activity and attenuated dopamine transporter function in the medial prefrontal cortex (mPFC) compared with isolated rats [21]. After repeated cocaine administration, conditioned place preference behavior developed, and c-Fos expression could be detected in the anterior cingulate cortex, the lateral CPu, the shell of the NAc, the dentate gyrus (DG) of the hippocampus, the basolateral (BLA) and central amygdala (CeA), and the VTA; however, EE reduced cocaine-induced behavioral sensitization and c-Fos expression in these brain regions [19].

The present study examined the following aspects: (a) whether different EE conditions produced different changes in MAMPH-induced behavioral sensitization and anxiety behaviors; and (b) whether the mPFC (e.g., cingulate cortex 1 (Cg1), prelimbic cortex (PrL), and infralimbic cortex (IL)), BLA, NAc, VTA, CPu, or hippocampus (e.g., CA1, CA3, and DG) are involved in the amelioration of behavioral sensitization under different EE conditions.

## 2. Material and Methods

### 2.1. Animals

Seventy-nine male C57BL/6J mice were purchased from the National Laboratory for Animal Breeding and Research Center, Taipei, Taiwan. At the beginning of the experiment, all mice weighed between 20 and 35 g. Mice were housed in groups in plastic cages with sawdust bedding in a colony room with temperature maintained at 23 ± 2 °C and a 12-h/12-h light/dark cycle (lights on: 07:00–19:00) with food and water available ad libitum. For the standard housing (SH, No EE), physical EE (PEE), and cognitive EE (CEE) groups, two mice were housed in each cage. For the social EE (SEE) and standard EE (STEE) groups, five mice were housed in each cage. All mice were subjected to behavioral tests; 4–5 mice were randomly selected for c-Fos immunohistochemical staining. All experiments were performed in accordance with the guidelines established by the American Psychological Association. Efforts were made to minimize both the number of animals used and the suffering of the animals.

### 2.2. Apparatus

#### Open-Field Task

The open-field task is performed in a square plastic box (50 cm × 50 cm × 38 cm). Each side of the square is equally divided into four parts, resulting in the establishment of sixteen sub-squares [23]. The open-field test is designed to measure locomotion and anxiety behaviors, and mouse movements were recorded using video-tracking software (Video Tracking Record System Version 1.17, SINGA Technology Corporation, Taipei, Taiwan).

### 2.3. General Procedure

At the start of the experiment, all mice were allowed a 7-day adaptation period in the colony room (Days 1–7). After the adaptation period, mice were housed under different housing conditions throughout the entire experiment (Days 8–29). After that, all mice were subjected to MAMPH-treatment training, during which mice were administered an intraperitoneal injection of 1 mg/kg MAMPH or normal saline, followed by a 30-min recovery period in their home cages, after which they were placed in the open-field apparatus for 15 min. This procedure was repeated once per day for 7 continuous days (Days 15–21). For the following 7 days, constituting the withdrawal phase, the mice did not receive any drugs and remained in their home cages (Days 22–28). During the testing phase (Day 29), all mice were intraperitoneally injected with either 0.5 mg/kg MAMPH (MAMPH groups) or normal saline (normal saline group), allowed to recover in their home cages for 30 min, and were then placed in the open-field apparatus for 15 min to test locomotor activity and anxiety behaviors. In different EE treatments, mice were assigned to either the No EE/Saline (*n* = 15), No EE/MAMPH (*n* = 10), STEE/MAMPH (*n* = 15), PEE/MAMPH (*n* = 12), CEE/MAMPH (*n* = 12), or SEE/MAMPH (*n* = 15) group (Figure S1).

### 2.4. Housing Styles and Environmental Enrichment

In the present study, the housing styles were determined according to the specific enrichment conditions being examined, including SH (No EE), STEE, PEE, CEE, and SEE. EE components can include many materials, such as a plastic ball, a tunnel, a wheel, a shelter, a rotating movable toy, stairs, a seesaw (Figure S2A), and companions. The components included in each cage differed according to the different EE conditions. The various EE designs are described in the following sections (Figure S2B–F).

#### 2.4.1. Standard Housing

SH (No EE) did not include any toys, running wheels, or shelters. However, the mice were housed with one companion in the home cage (two mice total). The number of mice for each cage in SE was based on previous literature [24]. The No EE group resided under SH conditions throughout the entire experiment. The cages were kept clean and tidy, and SH cages had only one floor. The floor area of SH cages was 28 cm long × 17 cm wide, and the cage litter was changed every 3 days (Figure S2B).

#### 2.4.2. Standard Environmental Enrichment

STEE included a wheel, a seesaw, a shelter, a plastic ball, stairs, a tunnel, and a rotating movable toy. Mice were raised with four companions in the STEE cage (five mice total). The number of mice for each cage in STEE was based on previous literature [8]. Environmental manipulation included changing the positions of toys and shelters every 5 days, and the cages were kept clean and tidy. The cage litter was changed every 3 days. The STEE cages featured two floors. The bottom floor was 24.5 cm long × 18 cm wide, and the top floor was 18 cm long × 10 cm wide. The running wheel was cleaned once per day (Figure S2C). This design was based on the description from a previous paper [8].

#### 2.4.3. Physical Environmental Enrichment

The design of PEE also included a wheel, a seesaw, a shelter, a plastic ball, stairs, a tunnel, and a rotating movable toy. In contrast to STEE, PEE mice only had one companion in each cage (two mice total). The number of mice for each cage in PEE was based on previous literature [10]. Environmental manipulation included changing the positions of toys and shelters every 5 days. The environment was kept clean and tidy, with cage litter changed every 3 days. The PEE cage featured two floors. The bottom floor was 24.5 cm long × 18 cm wide, and the top floor was 18 cm long × 10 cm wide. The running wheel was cleaned once per day (Figure S2D). This design was modified from descriptions presented in previous studies [7,10].

#### 2.4.4. Cognitive Environmental Enrichment

CEE included a wheel, a seesaw, a shelter, a plastic ball, stairs, a tunnel, and a rotating movable toy. CEE focused on enriched environments without the inclusion of running wheels; however, mice in CEE cages were raised with only one companion (two mice total). The number of mice for each cage in CEE was based on a modification of previous literature [10]. In contrast to PEE, CEE did not have a running wheel, but CEE had the same number of companion as PEE. In CEE, environmental manipulation also included changing the positions of toys and shelters every 5 days. The environment was kept clean and tidy, and the cage litter was changed every 3 days. The CEE cages featured two floors. The bottom floor was 24.5 cm long × 18 cm wide, and the top floor was 18 cm long × 10 cm wide. CEE contained no running wheels (Figure S2E). This design was modified from a description presented in a previous publication [11].

#### 2.4.5. Social Environmental Enrichment

SEE did not include a wheel, a seesaw, a plastic ball, stairs, a tunnel, or a rotating movable toy. In contrast to other EEs, SEE focused on enhanced social interactions, and thus mice had four companions (five mice total) but were deprived of physical stimulation or toys. The number of mice for each cage in SEE was based on previous literature [25]. The environment was kept clean and tidy, and the cage litter was changed every 3 days. The SEE cage had only one floor, with a floor area sized 24.5 cm long × 18 cm wide. To prevent fighting among the mice, a shelter was placed in the SEE cage (Figure S2F). This design was modified from descriptions presented in previous papers [7,10].

### 2.5. Drug

MAMPH was purchased from the Pharmaceutical Plant of the Food and Drug Administration, Ministry of Health and Welfare, Executive Yuan (Taipei, Taiwan). Sodium chloride was purchased from Sigma-Aldrich (St. Louis, MO, USA). MAMPH was prepared in normal saline at a concentration of 1 mg/mL and administered intraperitoneally. Sodium chloride was dissolved in distilled water and prepared as a normal saline solution. The injection volumes used for both MAMPH and normal saline were 1 mL/kg. Doses of 1 mg/kg and 0.5 mg/kg MAMPH were used in the study. After completing the last behavioral test, immunohistochemical staining for c-Fos was performed in select brain areas, including the Cg1, PrL, IL, NAc, BLA, VTA, CPu, CA1, CA3, and DG.

### 2.6. Immunohistochemical Staining

One hundred twenty minutes after completing behavioral tests, all mice were sacrificed with an overdose of sodium pentobarbital by injection. Later, the mice were perfused with an appropriate volume of 0.1 M sodium phosphate-buffered saline (PBS) and then injected with 4% paraformaldehyde in a 0.1 M PBS buffer. After that, the brain tissues were post-fixed (1 day) and transferred to 30% sucrose for cryoprotection until they sank to the bottom of the solution. Using a freezing and sliding microtome, 40-μm slices were cut through the whole brain. All slices were subjected to c-Fos immunoreactivity. The free-floating slices were washed once for 15 min in 0.1 M PBS, permeabilized in 3% H2O2 for 1 h, washed in 2% PBS tween-20 (PBST) for 20 min, and soaked in 3% normal goat serum and 1% bovine serum albumin for 1 h. After completing PBST washes twice for 15 min, the slices were incubated at 4 °C overnight for labeling c-Fos proteins (rabbit anti-c-Fos primary antibody; Santa Cruz Biotechnology Inc., SC-52, 1:1000). Then, the slices were washed with the PBST buffer twice for 15 min and incubated 1 h with a biotinylated goat anti-rabbit secondary antibody (Vector Lab BA-1000, 1:500). The slices were washed with PBS buffer for 10 min. The brain slices were tested for the bound secondary antibody, and the immunoreactivity was amplified using the ABC kit (Vector Lab ABC Kit, PK-6100). Quantification of c-Fos entailed several steps. To quantify c-Fos expression in the PrL of the mPFC, we used lower magnification (×4) to find the reference brain area (e.g., forceps minor of the corpus callosum (fmi)). At the same time, we used the mouse brain atlas to check the PrL’s location with respect to the reference brain area (the fmi). After confirming the PrL’s location, we increased the magnification to ×20 to narrow down the view and then took a photo of the brain slice. After that, the positive expression of c-Fos proteins in the neuron was counted by ImageJ software at ×20 magnification for each slice of the specific brain area [26]. The c-Fos quantity of each slice in the specific brain area was averaged for every group.

### 2.7. Statistics

One-way analysis of variance (ANOVA) was performed to assess differences in the mean ± standard error of the mean (SEM) values between groups for behavioral test parameters, including total distance traveled (in mm), normalized time spent in the center of the open-field apparatus (%), and total distance traveled/distance traveled in the center. The normalized time spent in the center of the open-field apparatus (%) was calculated by the formula: 100 × (the time spent in center/total distance travelled). A higher score for normalized time spent in the center of the open-field apparatus (%) indicates higher anxiety response. A lower ratio of total distance traveled/distance traveled in the center indicates decreased anxiety behavior. The groups assessed were No EE/Saline (*n* = 15), No EE/MAMPH (*n* = 10), STEE/MAMPH (*n* = 15), PEE/MAMPH (*n* = 12), CEE/MAMPH (*n* = 12), and SEE/MAMPH (*n* = 15). Note that No EE represented SH housing. Moreover, c-Fos expression was analyzed by one-way ANOVA to examine differences in c-Fos expression across various brain areas, including Cg1, PrL, IL, NAc, BLA, VTA, CPu, CA1, CA3, and DG. Significant differences detected by one-way ANOVA were verified using post hoc Tukey’s test. Values of *p* < 0.05 were considered significant. Finally, combining the behavioral data and c-Fos data of all groups, Pearson correlation tests were conducted to test the correlation between total distance traveled and c-Fos expression in specific brain areas, including Cg1, PrL, IL, NAc, BLA, VTA, CPu, CA1, CA3, and DG.

## 3. Results

### 3.1. Environmental Enrichment: Locomotion and Anxiety Tests

**Figure 1:**
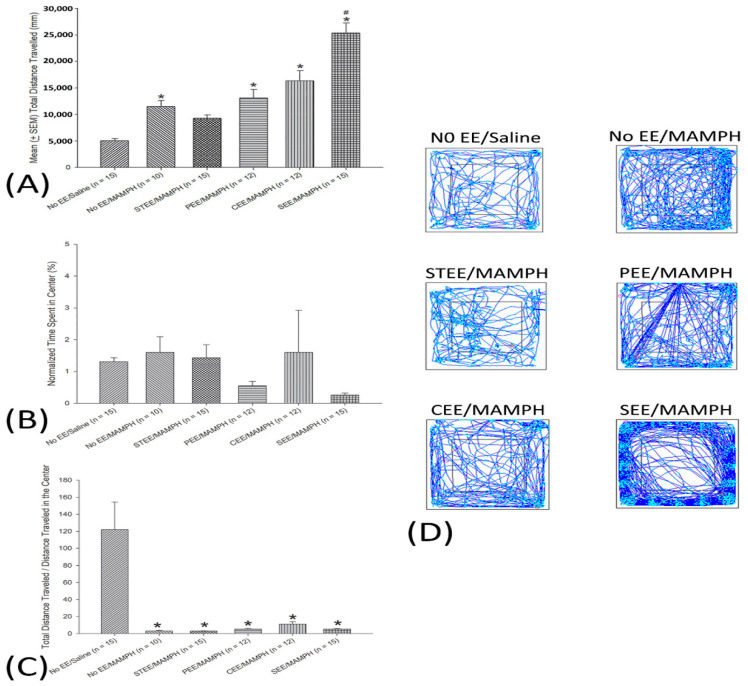
Various environmental enrichment (EE) housing conditions had differential effects on methamphetamine (MAMPH)-induced behavioral sensitization as assessed by: (**A**) total distance traveled (in mm), (**B**) normalized time spent in center (%), and (**C**) total distance traveled/distance traveled in the center. Mice reared under standard housing (No EE) or various EE conditions were intraperitoneally injected with 1 mg/kg of MAMPH or normal saline. No EE/Saline (*n* = 15), No EE/MAMPH (*n* = 10), standard EE (STEE)/MAMPH (*n* = 15), physical EE (PEE)/MAMPH (*n* = 12), cognitive EE (CEE)/MAMPH (*n* = 12), and social EE (SEE)/MAMPH (*n* = 15). (**D**) Path tracing for the No EE/Saline (*n* = 15), No EE/MAMPH (*n* = 10), STEE/MAMPH (*n* = 15), PEE/MAMPH (*n* = 12), CEE/MAMPH (*n* = 12), and SEE/MAMPH (*n* = 15) groups. * *p* < 0.05 compared with the No EE/Saline group, and # *p* < 0.05 compared with the No EE/MAMPH group.

To determine whether differences in EE impacted MAMPH-induced locomotor activity, one-way ANOVA was used to compare the mean ± SEM values for total distance traveled among groups. Groups were identified as a significant factor for total distance traveled (F(5, 73) = 29.71, *p* < 0.05). Post hoc Tukey’s tests indicated that the No EE/MAMPH, PEE/MAMPH, CEE/MAMPH, and SEE/MAMPH groups traveled significantly farther than the No EE/Saline group (*p* < 0.05); however, the STEE/MAMPH group was not significantly different from the No EE/Saline (*p* > 0.05) or No EE/MAMPH groups in total distance traveled (*p* > 0.05). This means that the distance traveled by the STEE/MAMPH group was midway between the No EE/MAMPH and No EE/Saline groups. The No EE/MAMPH and No EE/Saline groups were described as significantly different. The SEE/MAMPH group displayed a significant increase in the total distance traveled compared with the No EE/MAMPH group (*p* < 0.05; Figure 1A).

One-way ANOVA revealed nonsignificant group effects on anxiety tests as assessed by normalized time spent (%) in the center of the open-field apparatus (F(5, 73) = 1.10, *p* > 0.05) (Figure 1B).

One-way ANOVA was conducted for the ratio of total distance traveled/distance traveled in the center. The results show a significant difference based on group (F(5, 73) = 10.85, *p* < 0.05). Post hoc with Tukey tests indicated that the ratio of total distance traveled/distance traveled in the center was significantly decreased in the No EE/MAMPH, STEE/MAMPH, PEE/MAMPH, CEE/MAMPH, and SEE/MAMPH groups compared to the No EE/Saline group (*p* < 0.05); however, the STEE/MAMPH, PEE/MAMPH, CEE/MAMPH, and SEE/MAMPH groups had nonsignificant differences compared to No EE/MAMPH (*p* > 0.05). The results indicate that MAMPH likely induced lower anxiety behavior; moreover, none of the EE affected the lowered anxiety responses (Figure 1C).

Path tracing results indicated an increase in the time spent in the center of the open-field apparatus for the No EE/MAMPH, STEE/MAMPH, PEE/MAMPH, CEE/MAMPH, and SEE/MAMPH groups compared with the No EE/Saline group. Combined with the data of total distance traveled/distance traveled in the center, MAMPH administration might decrease anxiety behaviors, which cannot be defined as a simple result of hyperactivity. However, STEE, PEE, CEE, and SEE did not influence MAMPH-induced lowered anxiety behaviors (Figure 1D).

### 3.2. Environmental Enrichment and Neural Activity: c-Fos Labeling

To determine the neural substrates involved in MAMPH-induced behavioral sensitization and the amelioration of MAMPH-induced behavioral sensitization mediated by EE exposure, one-way ANOVA was used to compare differences in c-Fos expression levels among groups.

**Figure 2:**
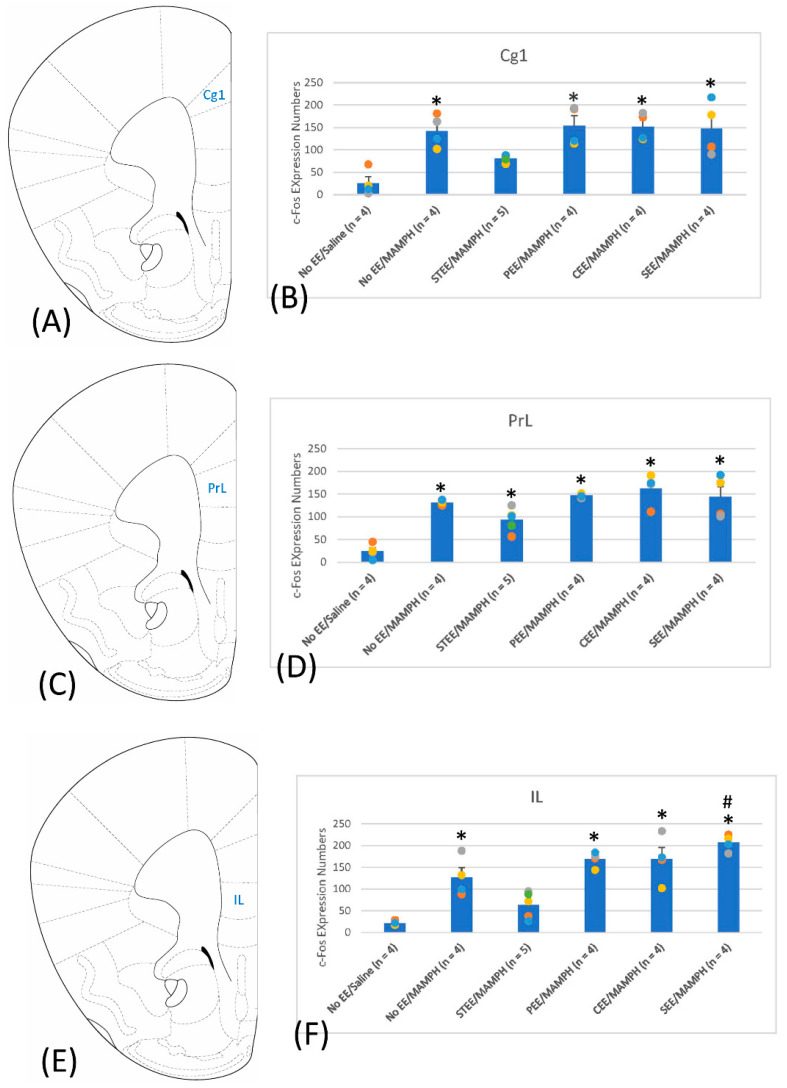
A cartoon brain atlas showing (**A**) the cingulate cortex 1 (Cg1), (**C**) prelimbic cortex (PrL), and (**E**) infralimbic cortex (IL) and c-Fos expression in (**B**) the Cg1, (**D**) PrL, and (**F**) IL for the No environmental enrichment (EE)/Saline (*n* = 4), No EE/methamphetamine (MAMPH; *n* = 4), standard EE (STEE)/MAMPH (*n* = 5), physical EE (PEE)/MAMPH (*n* = 4), cognitive EE (CEE)/MAMPH (*n* = 4), and social EE (SEE)/MAMPH (*n* = 4) groups; * *p* < 0.05 compared with the No EE/Saline group, and # *p* < 0.05 compared with the No EE/MAMPH group.

The results showed a significant group effect on c-Fos expression in the Cg1 (F(5, 19) = 8.38, *p* < 0.05) (Figure 2A,B and Figure S3A), PrL (F(5, 19) = 14.42, *p* < 0.05) (Figure 2C,D and Figure S3B), and IL (F(5, 19) = 19.25, *p* < 0.05) (Figure 2E,F and Figure S3C). Post hoc Tukey’s test indicated significantly increased c-Fos expression in the Cg1, PrL, and IL for the No EE/MAMPH group compared with the No EE/Saline group (*p* < 0.05), indicating that the Cg1, PrL, and IL of the mPFC may be involved in regulation of MAMPH-induced behavioral sensitization. Mice housed under STEE conditions showed significantly decreased c-Fos expression in the Cg1 and IL (*p* < 0.05) but not in the PrL (*p* > 0.05) compared with levels in the No EE/MAMPH group. Mice housed under SEE conditions showed significantly increased c-Fos expression in the IL (*p* < 0.05) but not in the C1g and PrL (*p* > 0.05). Mice housed under other EE conditions, including PEE and CEE, showed no significant differences in c-Fos expression in the Cg1, PrL, and IL compared with the No EE/MAMPH group (*p* > 0.05), indicating that PEE and CEE conditions did not change c-Fos expression underlying MAMPH-induced behavioral sensitization in the Cg1, PrL, and IL (Figure 2).

**Figure 3:**
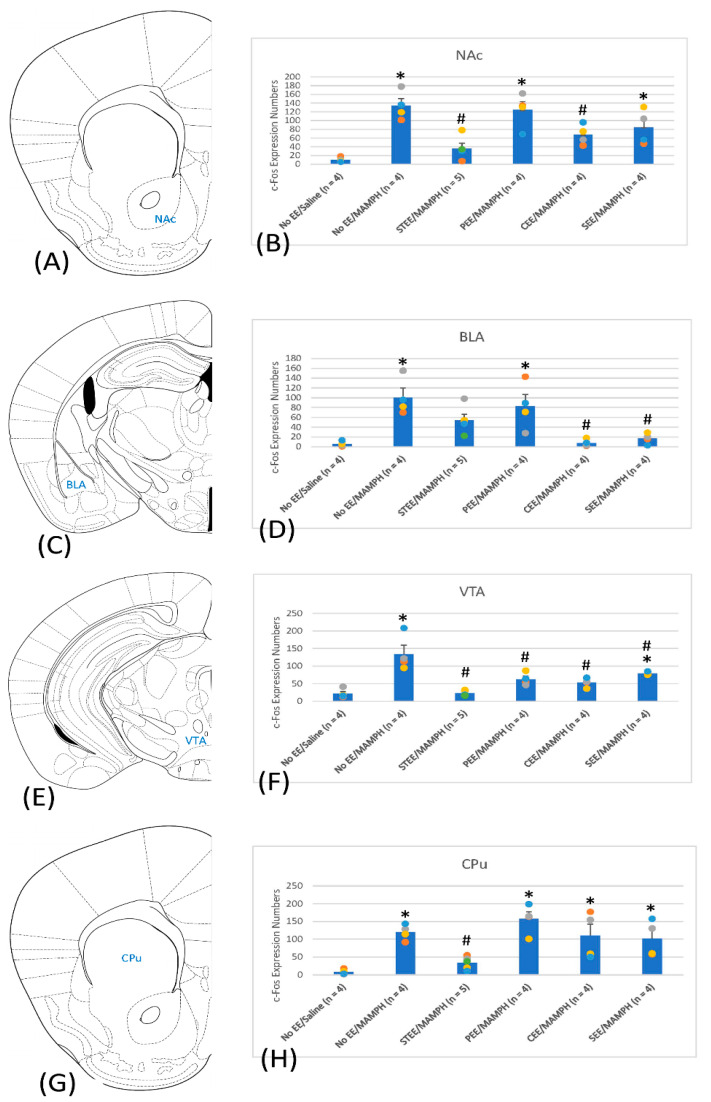
A cartoon brain atlas showing (**A**) the nucleus accumbens (NAc), (**C**) basolateral amygdala (BLA), (**E**) ventral tegmental area (VTA), and (**G**) caudate-putamen (CPu) and c-Fos expression in (**B**) the NAc, (**D**) BLA, (**F**) VTA, and (**H**) CPu for the No environmental enrichment (EE)/Saline (*n* = 4), No EE/methamphetamine (MAMPH; *n* = 4), standard EE (STEE)/MAMPH (*n* = 5), physical EE (PEE)/MAMPH (*n* = 4), cognitive EE (CEE)/MAMPH (*n* = 4), and social EE (SEE)/MAMPH (*n* = 4) groups; * *p* < 0.05 compared with the No EE/Saline group, and # *p* < 0.05 compared with the No EE/MAMPH group.

Comparisons of c-Fos expression in the NAc, BLA, VTA, and CPu revealed significant group effects in the NAc (F(5, 19) = 10.79, *p* < 0.05) (Figure 3A,B and Figure S4A), BLA (F(5, 19) = 8.63, *p* < 0.05) (Figure 3C,D and Figure S4B), VTA (F(5, 19) = 14.25, *p* < 0.05) (Figure 3E,F and Figure S4C), and CPu (F(5, 19) = 8.89, *p* < 0.05)] (Figure 3G,H and Figure S4D). Post hoc Tukey’s tests indicated a significant increase in c-Fos expression for the No EE/MAMPH group compared with the No EE/Saline group in the NAc, BLA, VTA, and CPu (*p* < 0.05), suggesting that NAc, BLA, VTA, and CPu were involved in MAMPH-induced behavioral sensitization. The STEE/MAMPH and CEE/MAMPH groups showed significantly decreased c-Fos expression in the NAc (*p* < 0.05) compared with that in the No EE/MAMPH group, indicating that STEE and CEE conditions decreased c-Fos expression induced by MAMPH in the NAc (Figure 3B). The CEE/MAMPH and SEE/MAMPH groups showed significantly decreased c-Fos expression in the BLA compared with that in the No EE/MAMPH group (*p* < 0.05), and no significant differences in c-Fos expression in the BLA were observed for the STEE/MAMPH group compared with the No EE/Saline and No EE/MAMPH groups (*p* > 0.05). These findings indicate that STEE, CEE, and SEE might reduce c-Fos expression induced by MAMPH in the BLA (Figure 3D). The results showed that c-Fos expression significantly decreased in the VTA for the STEE/MAMPH, PEE/MAMPH, CEE/MAMPH, and SEE/MAMPH groups compared with that in the No EE/MAMPH group (*p* < 0.05), indicating that STEE, PEE, CEE, and SEE decreased c-Fos expression in the VTA (Figure 3F). Only the STEE/MAMPH group showed significantly decreased c-Fos expression in the CPu (*p* < 0.05) compared with that in the No EE/MAMPH group, indicating that STEE ameliorated MAMPH-induced c-Fos expression in the CPu (Figure 3H).

**Figure 4:**
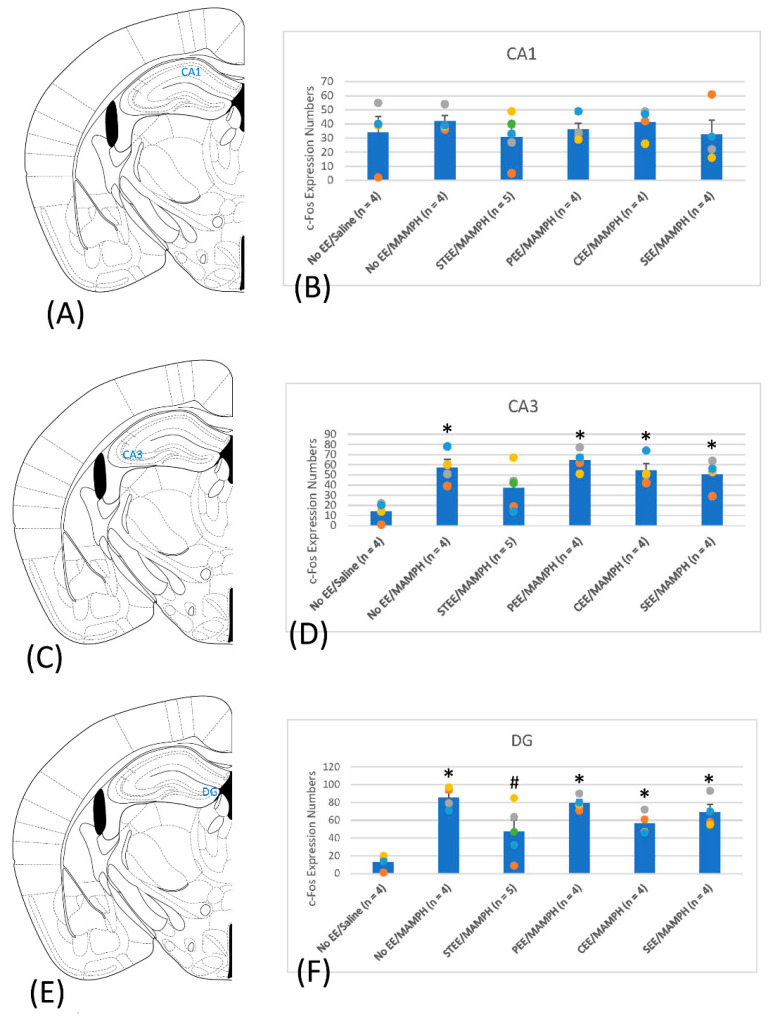
A cartoon brain atlas showing the (**A**) CA1, (**C**) CA3, and (**E**) dentate gyrus (DG) of the hippocampus and c-Fos expression in (**B**) the CA1, (**D**) CA3, and (**F**) DG for the No environmental enrichment (EE)/Saline (*n* = 4), No EE/methamphetamine (MAMPH; *n* = 4), standard EE (STEE)/MAMPH (*n* = 5), physical EE (PEE)/MAMPH (*n* = 4), cognitive EE (CEE)/MAMPH (*n* = 4), and social EE (SEE)/MAMPH (*n* = 4) groups; * *p* < 0.05 compared with the No EE/Saline group, and # *p* < 0.05 compared with the No EE/MAMPH group.

The results reveal significant group effects on MAMPH-induced c-Fos expression for the CA3 (F(5, 19) = 5.56, *p* < 0.05) (Figure 4C,D and Figure S5B) and DG (F(5, 19) = 9.54, *p* < 0.05) (Figure 4E,F and Figure S5C) but not for the CA1 (F(5, 19) = 0.36, *p* > 0.05) (Figure 4A,B and Figure S5A). Post hoc Tukey’s tests indicated a significant increase in c-Fos expression for the No EE/MAMPH compared with the No EE/Saline group in the CA3 and DG (*p* < 0.05) but not in the CA1 (*p* > 0.05), suggesting that the CA3 and DG (but not the CA1) are involved in MAMPH-induced behavioral sensitization. The STEE/MAMPH group showed no significant differences in c-Fos expression in the CA3 compared with the No EE/MAMPH and No EE/Saline groups (*p* > 0.05), indicating that STEE ameliorated MAMPH-induced c-Fos expression in the CA3 (Figure 4D). The STEE/MAMPH group showed significantly decreased c-Fos expression in the DG compared with the No EE/MAMPH group (*p* < 0.05), indicating that STEE ameliorated MAMPH-induced c-Fos expression in the DG (Figure 4F).

Concerning the correlation between total distance traveled and specific brain areas’ c-Fos expression. Pearson correlation tests showed that the total distance traveled was positively correlated with c-Fos expression in the Cg1 (r = 0.45, *p* < 0.05), PrL (r = 0.59, *p* < 0.05), IL (r = 0.62, *p* < 0.05), and CPu (r = 0.44, *p* < 0.05); however, total distance traveled showed nonsignificant correlation with c-Fos expression in the NAc (r = 0.03, *p* > 0.05), BLA (r = −0.20, *p* > 0.05), VTA (r = 0.14, *p* > 0.05), CA1 (r = 0.05, *p* > 0.05), CA3 (r = 0.24, *p* > 0.05), and DG (r = 0.14, *p* > 0.05; Table 1). Therefore, the results indicate that neuronal activity in the Cg1, PrL, IL, and CPu has positive correlation with MAMPH-induced behavioral sensitization in the total distance traveled.

## 4. Discussion

MAMPH induced behavioral sensitization, as indicated by increases in the total distance traveled. However, data from the current study did not change normalized time spent in the center of the open-field apparatus, indicating MAMPH did not induce anxiety behaviors. These findings suggest that MAMPH administration induced strong behavioral sensitization effects without inducing anxiety behavior. Mice housed under STEE conditions were likely to reduce MAMPH-induced behavioral sensitization, whereas mice housed under SEE conditions demonstrated an increase in MAMPH-induced behavioral sensitization.

The expression of c-Fos was examined in targeted regions that might represent neural substrates underlying the observed MAMPH-induced behavioral sensitization, and showed that the Cg1, PrL, and IL of the mPFC, the NAc, BLA, VTA, and CPu, and the CA3 and DG of the hippocampus might be involved in MAMPH-induced behavioral sensitization. STEE seemingly reduced MAMPH-induced behavioral sensitization and decreased c-Fos expression in the Cg1, IL, NAc, BLA, VTA, CPu, CA3, and DG. By contrast, SEE facilitated MAMPH-induced behavioral sensitization and changes in c-Fos expression in the IL, BLA, and VTA. In addition, decreased c-Fos expression was observed in the NAc, BLA, and VTA for CEE conditions and the VTA for PEE conditions; however, these changes were not associated with significant changes in behavioral sensitization.

### 4.1. Does Methamphetamine Induce Behavioral Sensitization and Comorbid Anxiety Behaviors?

The present results reveal that MAMPH increased the total distance traveled in the No EE/MAMPH group compared with the No EE/Saline group; however, MAMPH administration did not increase the normalized time spent in the center of the open-field apparatus compared with these values in the No EE/Saline group, indicating that MAMPH induced behavioral sensitization without inducing anxiety behaviors. The present data were partially aligned with previous data [13,14,27], which showed that amphetamine induced behavioral sensitization and stereotypical behaviors in deer mice [27]. EE has been shown to disrupt amphetamine-induced behavioral sensitization in locomotor activity without changing dopamine levels in the NAc and striatum [13]. Rats reared under EE conditions showed the attenuation of low-dose amphetamine-induced behavioral sensitization in locomotor activity compared with isolated and standard rearing conditions [14]. However, the present findings do not align with previous evidence that MAMPH injections induce anxiety behavior [28,29,30]. For example, a previous study showed that MAMPH administration increased rearing and locomotor activity in an open-field task and increased time spent in the center while decreasing time spent in the corners, indicating MAMPH simultaneously induced behavioral sensitization and anxiety behaviors [28]. Acute MAMPH administration resulted in decreased time spent in the center during an open-field task, indicating the development of anxiety behavior; however, voluntary exercise resulted in an increase in the time spent in the center, indicating that voluntary exercise was able to ameliorate MAMPH-induced anxiety behavior [29]. MAMPH-treated mice demonstrated increased locomotor activity and anxiety behaviors in the open-field task compared with the saline group in another study [30]. Further studies are necessary to understand why the present data are inconsistent with previous findings.

### 4.2. Effects of Modulating Environmental Enrichment Components on Behavioral Sensitization and Comorbid Anxiety Behaviors

Previously, no research had comprehensively examined how different EE modulated behavioral sensitization, with only a few studies examining different EE conditions [10,11]. For example, short-term CEE without exercise protocols was found to improve recognition and working memory and increase neuronal growth factors, such as neurotrophins, enhancing hippocampal neurogenesis [11]. Another study employed SEE and PEE to dissociate the effects of social behavior and cognition in neural reactivity [10]. In this study, the design of SEE consisted of rearing with five companions, whereas the design of PEE consisted of physically enriched housing conditions. The data show that PEE increased neurogenesis in the hippocampal DG but was less effective for improving social approach behaviors. By contrast, SEE was less effective for neuronal plasticity and cognition but enhanced pro-social 50-kHz ultrasonic vocalizations and socially relevant behaviors. Therefore, SEE and PEE appear to result in different effects for neural plasticity, cognition, and social behaviors [10].

The present study is the first to comprehensively examine the effects of different EE housing conditions (i.e., STEE, PEE, CEE, and PEE) on behavioral sensitization and anxiety behaviors. Our data suggest that STEE is the most appropriate EE condition to ameliorate MAMPH-induced behavioral sensitization, whereas SEE appeared to facilitate MAMPH-induced behavioral sensitization, based on the total distance traveled. Path tracing outcomes for the No EE/MAMPH group indicated general hyperactivity increased the time spent in the center and throughout the entire open-field apparatus. STEE significantly decreased MAMPH-induced hyperactivity. However, SEE appeared to enhance MAMPH-induced hyperactivity (Figure 1A), and mice in the SEE group likely spent a lot of time on the edges of the open-field apparatus (Figure 1D). However, SEE did not affect normalized time spent in the center (Figure 1B) and total distance traveled/distance traveled in the center (Figure 1C) compared to the EE/MAMPH group. Therefore, SEE seemingly facilitated behavioral sensitization induced by MAMPH but did not change MAMPH-induced lowered anxiety behaviors. Further studies should address the issue of whether SEE can decrease MAMPH-induced lowered anxiety behaviors. Moreover, further studies should consider testing stress hormone corticosterone levels in plasma following any EE and MAMPH injections. Stress biomarker such as corticosterone levels clarify the anxiety effect of MAMPH injections associated with different EE strategies.

On the other hand, STEE (but not other EE) might blunt MAMPH-induced behavioral sensitization, based on the assessment of locomotor activity, but STEE did not appear to decrease anxiety behavior (Figure 1B). The present results of STEE are not supportive of previous evidence [8]. For example, continuous exposures (but not intermittent exposures) to novel objects in STEE reduced amphetamine-induced behavioral sensitization and anxiety behaviors [8]. Alternatively, STEE includes all of the EE components, including sensory, physical, cognitive, and social stimulations, leading to the effective reduction of MAMPH-induced behavioral sensitization. These results were consistent with the previous findings that rats reared in EE conditions presented reduced behavioral sensitization based on locomotor activity compared with rats reared in isolation or under SH conditions (No EE) [9]. Therefore, this issue of whether STEE could decrease psychostimulant-induced behavioral sensitization and anxiety behaviors remains to be further investigated.

### 4.3. The Involvement of the Cg1, PrL, and IL in MAMPH-Induced Behavioral Sensitization under Different EE Conditions

The present study shows that the Cg1 and IL expressed seemingly reduced levels of c-Fos in the STEE/MAMPH group than in the No EE/MAMPH group, indicating the Cg1 and IL are likely involved in the effects of STEE in reducing MAMPH-induced behavioral sensitization. By contrast, SEE housing conditions appeared to enhance c-Fos expression compared with that in the No EE/MAMPH group, indicating that SEE increased neural activity associated with MAMPH-induced behavioral sensitization. No significant changes in c-Fos expression in the Cg1, PrL, and IL were observed in mice raised under other EE conditions compared with the No EE/MAMPH group. The present study findings do not fully align with previous findings [21,31,32,33]. One behavioral study showed that EE was able to disrupt the adverse effects associated with withdrawal from alcohol consumption, indicating that EE housing styles might protect the integrity of mPFC functions, including in the Cg1, PrL, and IL [33]. Acute but not chronic EE exposure was found to blunt cue-related and instrumental lever pressing for sucrose intake, associated with reduced c-Fos expression in the dorsal mPFC, including the PrL, but not in the IL [32]. Rats housed under isolated housing conditions and exposed to cocaine prenatally presented with divergent social interactions and changes in dopamine transporter functions; however, STEE housing conditions were found to attenuate the effects of prenatal cocaine administration on socially divergent behaviors, locomotor activity, and the dopamine clearance rate in the mPFC, indicating that EE interfered with behavioral and neurochemical effects induced in the mPFC by prenatal cocaine administration [21]. Opposing evidence indicates that early EE exposure decreased neuronal apoptosis, synaptic proteins loss, myelination defects, and microglial activation in the hippocampus but not in the mPFC [31].

In conclusion, STEE involved all components of housing stimuli, and blunted behavioral sensitization; moreover, it decreased c-Fos expression in the Cg1 and IL but not the PrL. SEE oppositely enhanced c-Fos expression in the IL, but the other EE strategies did not change c-Fos expression in the sub-regions of the mPFC. Seemingly, the variations in EE housing designs might be associated with inconsistent results among regions of the mPFC. Some questions emerged as to whether the sub-regions of the mPFC (e.g., Cg1, PrL, and IL) under the different EE conditions play different roles in modulating MAMPH-induced behavioral sensitization; moreover, the Cg1 and IL of the mPFC downregulated c-Fos expression and neural activity, reducing behavioral sensitization using STEE. These issues should be investigated in future studies. The present findings might offer some clinical implications.

### 4.4. The Involvement of the VTA, NAc, BLA, and CPu in MAMPH-Induced Behavioral Sensitization under Different EE Housing Conditions

Considerable research indicates that the VTA, NAc, BLA, and CPu are likely to contribute to EE-associated modulations of reward-related and addictive behaviors [19,34,35]. For example, isolated housing was shown to facilitate cue-elicited cocaine self-administration behavior, whereas EE housing reduced cue-induced drug self-administration behavior. Moreover, under EE housing conditions, reduced c-Fos expression was detected in the PrL, IL, anterior cingulate cortex, NAc shell and core, orbitofrontal cortex, and BLA [35]. A similar study demonstrated that EE conditions attenuated cocaine-induced conditioned place preference behavior and neuronal activity, indicated by decreased c-Fos expression in the anterior cingulate cortex, lateral CPu, NAc shell, DG, BLA, CeA, and VT [19]. A study of neuroinflammation revealed that EE attenuated lipopolysaccharide-induced sickness behavior, which was associated with reduced IL-6 levels in the VTA [36]. Another study demonstrated that postnatal EE exposure ameliorated prenatal alcohol exposure-induced microglial morphology changes and disruptions in dopaminergic neuronal morphology in the VTA [37]. The present study appears to support these previous findings, revealing that STEE housing conditions resulted in significantly decreased c-Fos expression in the NAc, BLA, VTA, and CPu, indicating that these brain areas are likely involved in the STEE-mediated reduction in MAMPH-induced behavioral sensitization. SEE housing conditions decreased c-Fos expression in the BLA and VTA, indicating these two brain areas regulate SEE-induced enhanced behavioral sensitization by MAMPH.

### 4.5. The Involvement of the Hippocampal CA1, CA3, and DG Regions in MAMPH-Induced Behavioral Sensitization under Different EE Conditions

The present study observed reduced c-Fos expression in the hippocampal CA3 and DG but not the CA1 regions in mice housed under EE conditions compared with those in the No EE/MAMPH group, which is consistent with recent studies [22,38]. For example, EE diminished toluene-induced behavioral sensitization and increased dopamine D1 receptor expression in the hippocampus, prefrontal cortex, NAc, and caudate [22]. EE exposure also decreased MAMPH-induced self-administration behaviors and alterations in glucocorticoid receptors in the ventral and dorsal hippocampus [38]. Therefore, the hippocampus, especially the CA3 and DG, appears to play a crucial role in the EE-mediated regulation of reward learning and addictive behaviors.

### 4.6. Emerging Issues and Future Studies

A recent review paper reported that EE ameliorated drug addiction craving, relapse, reinstatement behaviors in preclinical studies and clinical work [39]. Moreover, the clinical work showed that EE reduced drug addiction by decreasing reward effect of abused drugs and cue association with drugs, and through relapse prevention [39]. From a clinical standpoint, EE design would include excellent social interaction, vocational training, physical exercise, and recreational and community involvement [39]. Furthermore, EE can cure various pathological and neurodegenerative diseases, including mental illness, cancer, traumatic brain injury, neurodegenerative diseases, and epilepsy in animal models [40]. Therefore, the role that different components of EE play in modulating various symptoms of pathological diseases needs to be examined further.

In light of previous data, MAMPH might induce rewarding or aversive effects based on dose [3,41]. Moreover, the reward due to or aversion of MAMPH is specific to sex [3,41]. For example, sex- and age-specific expressions of D1 and D2 are altered by chronic MAMPH (3 mg/kg) administrations; moreover, female mice were less sensitive to reward and showed less MAMPH-induced conditioned place preference compared to male mice [41]. After MAMPH (0.1 mg/kg) administration, females were more hyperactive in locomotor activity than males. However, female mice showed less conditioned place aversion than male mice. This indicates that females have resistance to the aversive effects of MAMPH [3]. Therefore, sexual differences are a crucial factor for examining the effect of MAMPH, regardless of reward or aversion. The present study only used male mice to determine how different EE affected MAMPH-induced behavioral sensitization. Differences in female mice is a limitation of the present study. These issues should be investigated in further studies.

The study did not include having each of the EE groups treated with saline. Instead, the study designed the No EE/Saline and EE/MAMPH groups to be the negative and positive controls, and the STEE, PEE, CEE, and SEE with MAMPH injections groups were compared with the No EE/Saline and No EE/MAMPH groups. The best condition is to design each EE/MAMPH group control. For example, the STEE/MAMPH compared with the STEE/Saline group; the PEE/Saline group compared with the PEE/MAMPH; the CEE/Saline group compared with the CEE/MAMPH; SEE/Saline group compared with the SEE/MAMPH. This is a limitation of the study and prevents us from determining whether the behavioral and c-Fos data are caused by a synergistic effect of MAMPH treatment and EE and/or what the individual contribution of EE has on these measures. This limitation should be prevented in future studies.

Analyzing Pearson correlation tests for total distance traveled and c-Fos expression in the specific brain areas showed that c-Fos expression in the Cg1, PrL, IL, and CPu was positively correlated with the total distance traveled. Some issues have emerged: whether the mPFC’s Cg1, PrL, and IL contribute different roles in modulating MAMPH-induced behavioral sensitization; moreover, the disparate EE conditions affect other effects of MAMPH-induced behavioral sensitization in the neuronal activity of the Cg1, PrL, and IL. Finally, the CPu is essential for motor functions. It should be examined how the CPu regulates MAMPH-induced behavioral sensitization and different EE strategies for hyperactivity. These issues warrant further concern.

In the present study, the software ImageJ counted c-Fos expression in specific brain areas. In the process of c-Fos expression quantification, the potential for bias should be discussed. For example, the labeled c-Fos proteins of the brain slice were loaded into the ImageJ program. The c-Fos protein image of the slice would be transformed into an RBG stake in red color. Then, the experimenter can adjust the threshold between the background and the c-Fos protein. Accordingly, bias potentially occurs during adjustment so that the threshold selected affected the accuracy of the quantification of c-Fos expression. These are the experimental limitations. To prevent this bias, quantification analysis of c-Fos expression can be considered by adding other blinders to count c-Fos expression, or counting can be double-checked and the data averaged. The potential for bias during quantification must be considered.

## 5. Conclusions

MAMPH induced behavioral sensitization as measured by total distance traveled, but did not appear to induce comorbid anxiety behavior. After MAMPH injections, mice presented with increased locomotor activity without evidence of anxiety during the open-field task. STEE included sensory, physical, and cognitive stimulation and was able to effectively reduce behavioral sensitization induced by MAMPH. Other EE conditions, including PEE, CEE, and SEE, were not able to effectively reduce MAMPH-induced behavioral sensitization, and SEE conditions appeared to facilitate behavioral sensitization, as measured by total distance traveled. These results are likely associated with an increase in stress associated with SEE housing conditions. The expression of c-Fos indicated that the Cg1, PrL, IL, NAc, BLA, VTA, CPu, CA3, and DG (but not the CA1) are likely to be involved in MAMPH-induced behavioral sensitization. The Cg1, IL, NAc, BLA, VTA, CPu, CA3, and DG regulated the STEE-mediated modulations of MAMPH-induced behavioral sensitization. Our findings regarding c-Fos expression and behavioral sensitization in response to different EE conditions may have clinical implications. The present data reveal that different EE conditions have differential effects on MAMPH-induced behavioral sensitization and neuronal activity in associated brain areas, which may inform the development of non-pharmacological treatments in addition to conventional pharmacological interventions, leading to the development of novel treatments for MAMPH addiction in the future.

## Figures and Tables

**Table 1:** Pearson correlation tests between total distance traveled (mm) and c-Fos expression in specific brain areas, including the Cg1, PrL, IL, NAc, BLA, VTA, CPu, CA1, CA3, and DG.

	Cg1	PrL	IL	NAc	BLA	VTA	CPu	CA1	CA3	DG
Total Distance traveled	r = 0.45, *p* < 0.05 *	r = 0.59, *p* < 0.05 *	r = 0.62, *p* < 0.05 *	r = 0.03, *p* > 0.05	r = −0.20, *p* > 0.05	r = 0.14, *p* > 0.05	r = 0.44, *p* < 0.05 *	r = 0.05, *p* > 0.05	r = 0.24, *p* > 0.05	r = 0.14, *p* > 0.05

(*) *p* < 0.05 significant differences; Cg1: cingulate cortex 1; PrL: prelimbic cortex; IL: infralimbic cortex; NAc: nucleus accumbens; BLA: basolateral amygdala; VTA: ventral tegmental area; CPu: caudate-putamen; DG: dentate gyrus.

## Data Availability

Data Availability Statements at https://www.dropbox.com/sh/02f8ukbifht18hl/AAA1qpdu8hb31eJpnRyCAo6Ma?dl=0 (accessed on 3 April 2022).

## Supplementary Materials

The following supporting information can be downloaded at: https://www.mdpi.com/article/10.3390/jcm11113051/s1, Figures S1–S5 and Supplementary caption.
