# Supplementary material for: Environmental Enrichment Components Required to Reduce Methamphetamine-Induced Behavioral Sensitization in Mice: Examination of Behaviors and Neural Substrates
Source: J Clin Med. 2022 May 28;11(11):3051. doi: 10.3390/jcm11113051 (PMC9181252; doi:10.3390/jcm11113051)
Supplement: Supplementary file 1 [file jcm-11-03051-s001.zip › jcm-1689862-supplementary.pdf]

Supplementary Materials and Methods

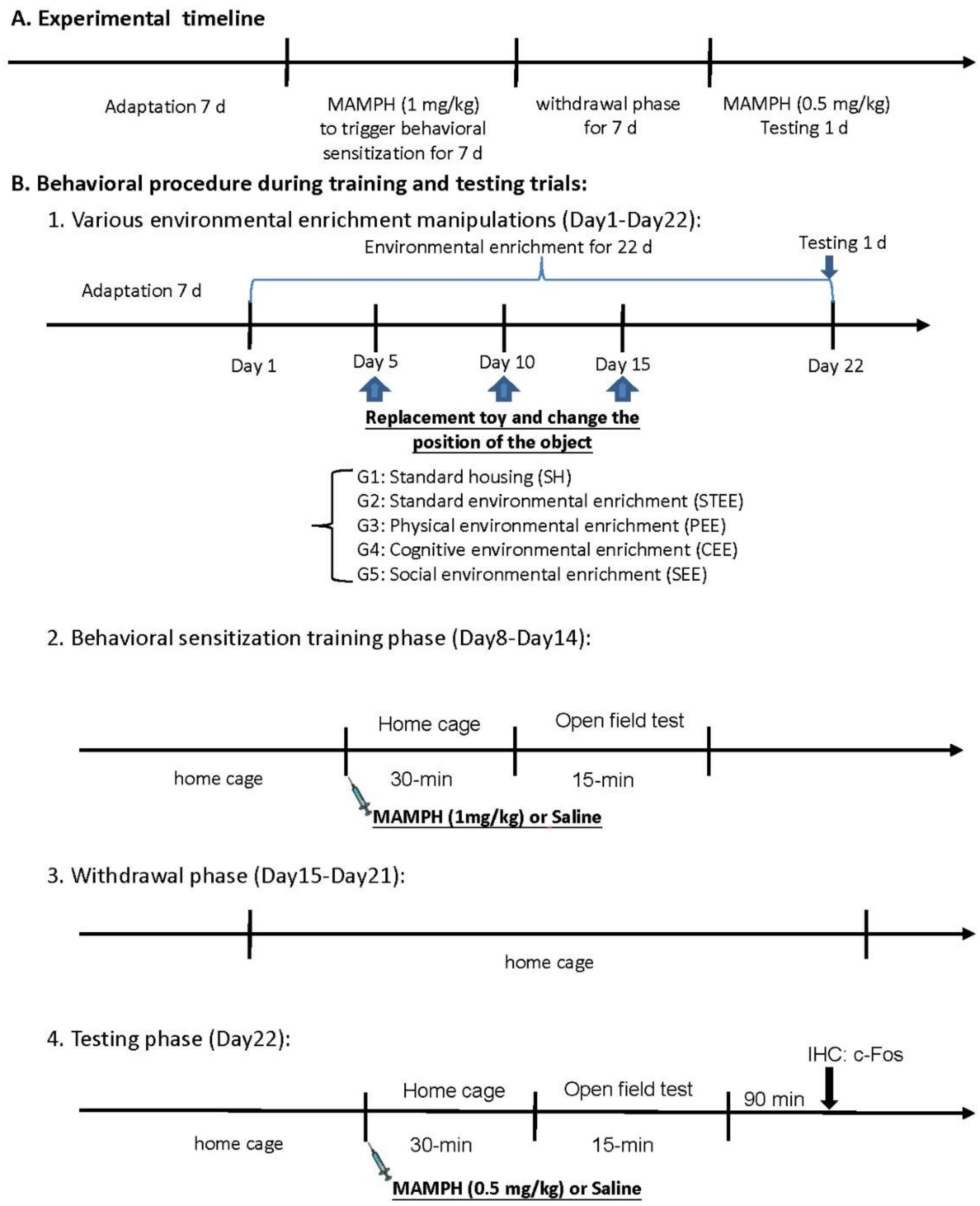

Figure S1. Overview of the experimental procedures.

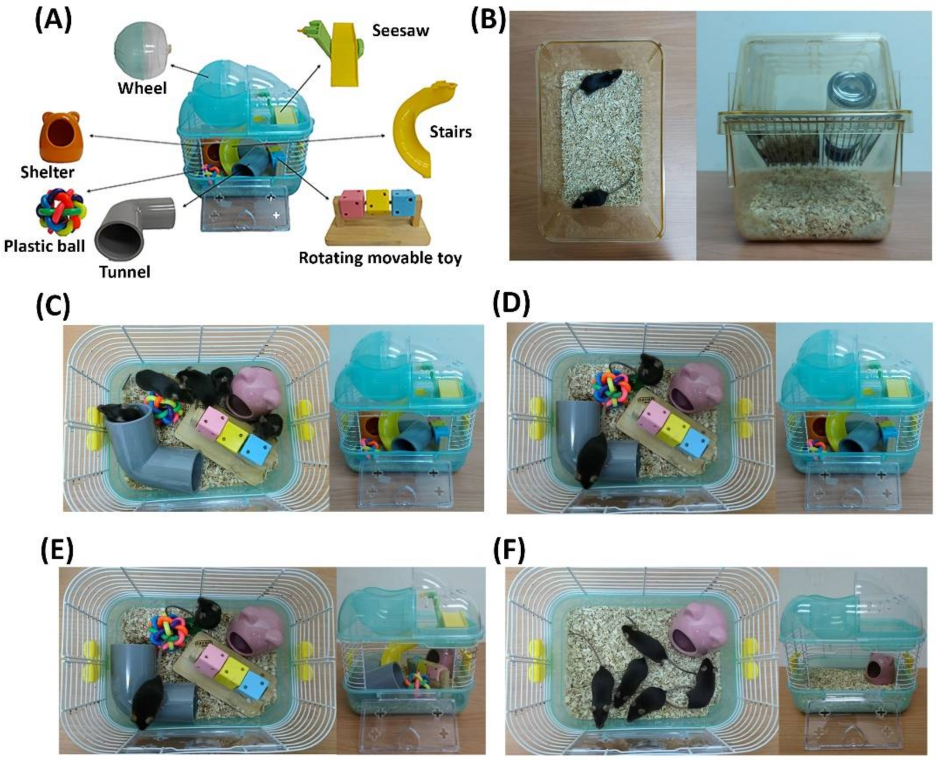

Figure S2. depicts (A). The components included in environmental enrichment(EE) housing designs. EE includes a wheel, a seesaw, a shelter, a plastic ball, stairs, a tunnel, and a rotating movable toy.The differentenvironmental enrichment (EE) housing styles,including (B) standard housing, (C) standard EE, (D) physical EE, (E) cognitive EE, and (F) social EE.

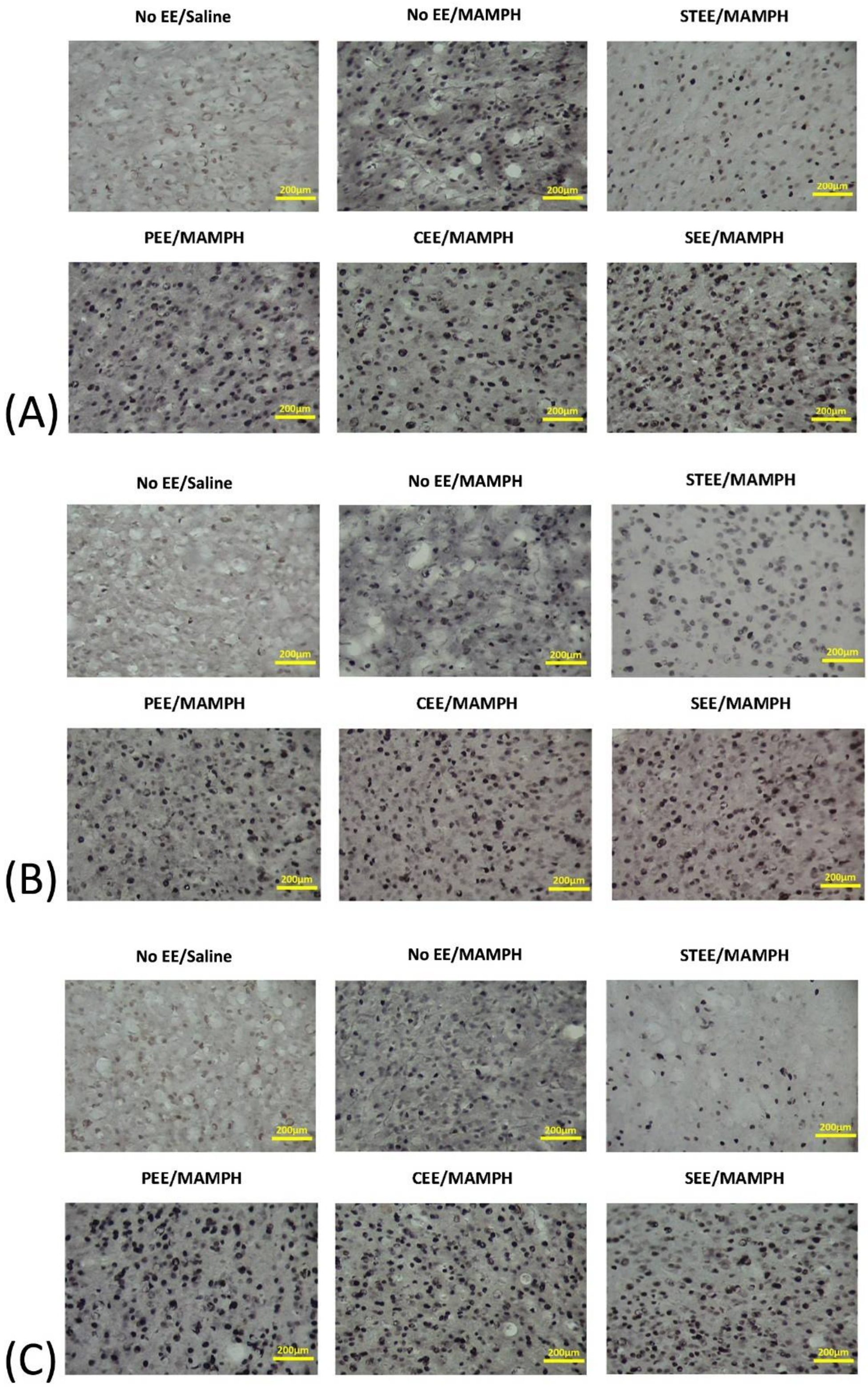

Figure S3. Representative photomicrographs of c-Fos expression for (A) the Cg1, (B) PrL, and (C) IL in the No EE/saline, No EE/MAMPH, STEE/MAMPH, PEE/MAMPH, CEE/MAMPH, and SEE/MAMPH groups. Scale bar represents 200 μm.

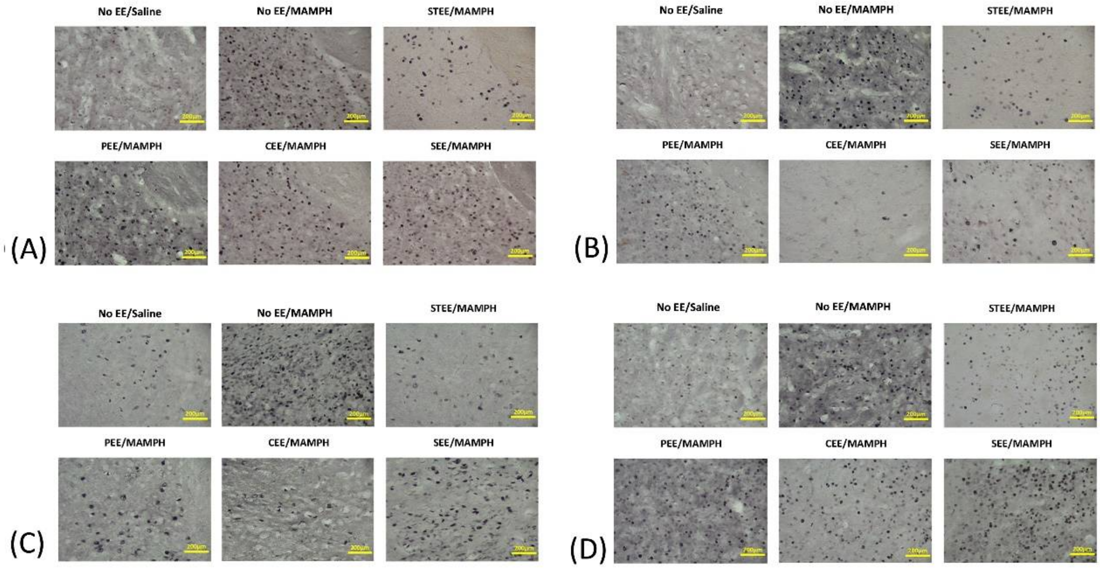

Figure S4. Representative photomicrographs of c-Fos expression for (A) the NAc, (B) BLA, (C) VTA, and (D) CPu the No EE/saline, No EE/MAMPH, STEE/MAMPH, PEE/MAMPH, CEE/MAMPH, and SEE/MAMPH groups. Scale bar represents 200 μm.

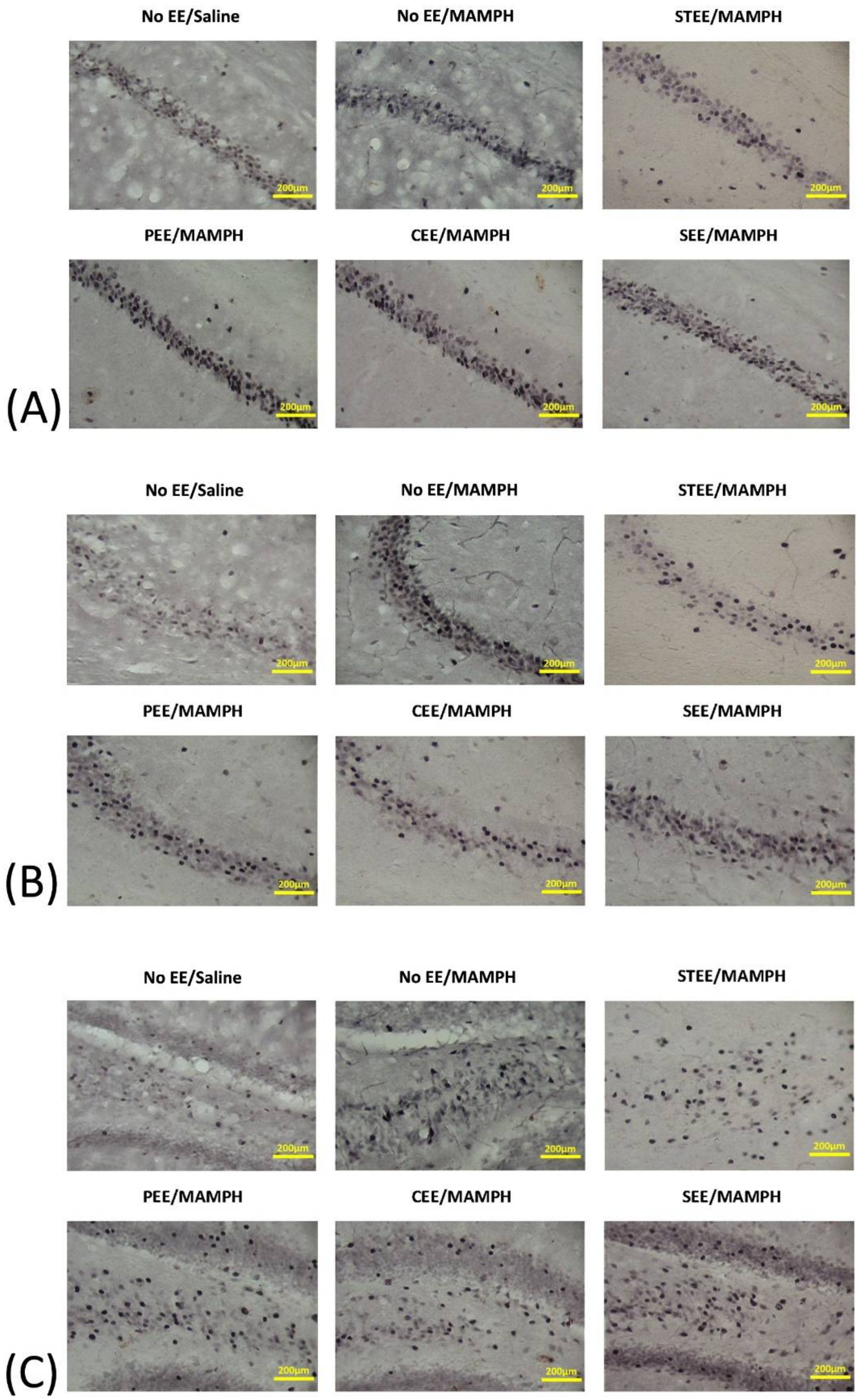

Figure S5. Representative photomicrographs of c-Fos expression for (A) the CA1, (B) CA3, and (C) DG in the No EE/saline, No EE/MAMPH, STEE/MAMPH, PEE/MAMPH, CEE/MAMPH, and SEE/MAMPH groups. Scale bar represents 200 μm.
